# 5D Parameter Estimation of Near-Field Sources Using Hybrid Evolutionary Computational Techniques

**DOI:** 10.1155/2014/310875

**Published:** 2014-02-18

**Authors:** Fawad Zaman, Ijaz Mansoor Qureshi

**Affiliations:** ^1^Department of Electronic Engineering, IIU, H-10, Islamabad 44000, Pakistan; ^2^Electrical Department, Air University, Islamabad 44000, Pakistan

## Abstract

Hybrid evolutionary computational technique is developed to jointly estimate the amplitude, frequency, range, and 2D direction of arrival (elevation and azimuth angles) of near-field sources impinging on centrosymmetric cross array. Specifically, genetic algorithm is used as a global optimizer, whereas pattern search and interior point algorithms are employed as rapid local search optimizers. For this, a new multiobjective fitness function is constructed, which is the combination of mean square error and correlation between the normalized desired and estimated vectors. The performance of the proposed hybrid scheme is compared not only with the individual responses of genetic algorithm, interior point algorithm, and pattern search, but also with the existing traditional techniques. The proposed schemes produced fairly good results in terms of estimation accuracy, convergence rate, and robustness against noise. A large number of Monte-Carlo simulations are carried out to test out the validity and reliability of each scheme.

## 1. Introduction

Parameter estimation of signals is one of the key issues in array signal processing, which has direct applications in radar, sonar, seismic exploration, electronic surveillance, and so forth [1]. In the literature, various algorithms are available to discuss this issue, such as the MUSIC algorithm [2], the maximum likelihood (ML) algorithm [3], the matrix pencil (MP) algorithm [4], and the ESPRIT algorithm [5]. Many of these algorithms make a supposition that the sources are positioned in the far field of sensors array so that the signal received from them can be taken as plane waves. With this supposition, the wave front of each signal is only a function of the DOA of the sources, which is easy to deal with. However, the situation becomes complicated if the sources are situated closer to the sensor array (near field). In this case, the waves are considered to be spherical, where the wave-front of each signal is the function of DOA, as well as, range of the sources [6].

Many classical algorithms are also available to discuss the problem of near-field source localization, such as the linear prediction algorithm [7], the 2D MUSIC algorithm [8], and the ESPRIT based algorithms [9, 10]. However, these algorithms mainly focus on 2D case, that is, estimation of the elevation angle and range parameters. Some algorithms are also available which deal with the 3D case (elevation angle, azimuth angle, and range) of near-field sources, for example, [11–14]. In [11] expectation-maximization (EM) algorithm is proposed, but it suffers from heavy computations and iterative process. A unitary ESPRIT algorithm is developed in [12] which requires further parameter pairing process, while the algorithm presented in [13] heavily relies on different carrier frequencies and approximated sinusoidal signals and also requires high sampling narrow band data. A spectral search based method is presented in [14] which can only be used for underwater environment. In [15], comparatively an efficient algorithm based on cumulants is proposed for 4D parameter estimation of near-field sources (frequency, range, and 2D DOA), but it also requires a large number of snapshots and ends up with higher mean square error (MSE). Moreover, it is also unable to estimate the amplitude of signals.

Now to estimate the parameters of near-field sources, heuristic techniques like evolutionary computing techniques (ECT) can also be used in the field of optimization. ECT, which is also known as computational intelligence, is a subfield of artificial intelligence that can be employed for combinatorial as well as for continuous optimization problems. ECT has stochastic or metaheuristic optimization nature and is considered to be global optimization methods. These techniques include genetic algorithm (GA) [16], particle swarm optimization (PSO) [17], and differential evolution (DE) [18]. These techniques are based on the principle of biological evolution, such as genetic inheritance and natural selection. One of the most important features of ECT is that they become even more reliable and effective when hybridized with any other efficient scheme such as pattern search (PS), active set (AS), and interior point algorithm (IPA) [19–24].

In this paper, 5D parameters (amplitude, frequency, range, elevation angle, and azimuth angle) of near-field sources impinging on centrosymmetric cross array are jointly estimated. Initially we used GA, PS, and IPA alone, but then we adopt hybrid evolutionary computing techniques based on GA hybridized with PS or IPA. In these hybrid approaches, the solution starts with a global optimizer (GA) and ends up with local optimizers (PS or IPA). For this a new multiobjective fitness function is used, which is the combination of MSE and correlation between normalized desired and estimated vectors. It requires only a single snapshot, which obviously decreases the computational cost. The performances of these two hybrid approaches (GA-PS and GA-IPA) are compared not only with each other, but also with the individual performance of GA, IPA, and PS. Besides, the proposed hybrid schemes are also compared with the traditional techniques available in the literature [15].

Throughout the paper, matrices and vectors are represented by bold upper and lower case letters, respectively, whereas *T*, *H*, and *N* are used, respectively, for transpose, hermitian, and normalization of vectors or matrix.

The rest of the work is organized as follows. In Section 2, data model is developed for near-field sources, while Section 3 describes the signal subspace dimension. The proposed schemes are given in Section 4, while results and simulations are provided in Section 5. Finally, conclusion and future work direction are given in Section 6.

## 2. Signal Model for Near-Field Sources

**Figure 1:**
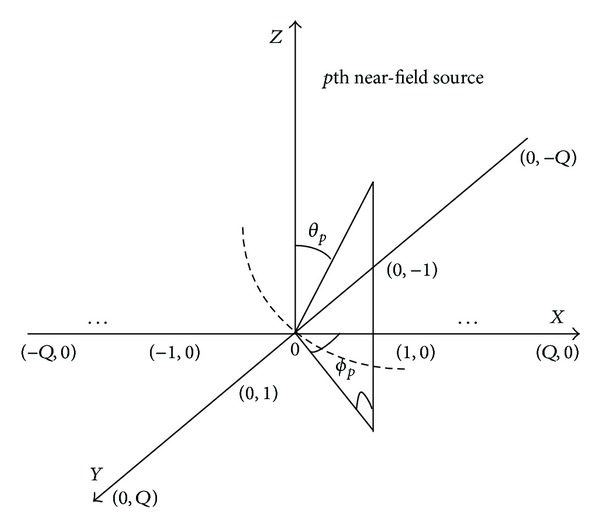
Signal model for near-field sources.

In this section, signal model for near-field sources impinging on centrosymmetric cross array (CSCA) is developed. All sources are considered to be narrow band and mutually statistically independent. The amplitude (*a*), frequency (*f*), range (*r*), and 2D DOA (*θ*, *ϕ*) are different for different sources. The CSCA is composed of two subarrays that are placed along *x*-axis and *y*-axis, respectively, as shown in Figure 1. The total number of sensors in the array is 4*Q* + 1 where each subarray consists of 2*Q* sensors, while the reference sensor is common among both. If *P* is the total number of sources, then the signal received at *m*th and *n*th sensor in *x*-axis and *y*-axis subarrays, respectively, can be modeled as

(1) $$\begin{array}{c} w_{m,0}=\overset{P}{\underset{p=1}{\sum }}a_{p}e^{j(m\alpha_{xp}+m^{2}\beta_{xp})}+\eta_{m,0}, \end{array}$$

(2) $$\begin{array}{c} w_{0,n}=\overset{P}{\underset{p=1}{\sum }}a_{p}e^{j(n\alpha_{yp}+n^{2}\beta_{yp})}+\eta_{0,n}, \end{array}$$

where *η*_*m*,0_ and *η*_0,*n*_ represent the additive white Gaussian noise (AWGN) added at *m*th and *n*th sensors in *x*-axis and *y*-axis subarrays, respectively.

In (1), *α*_*xp*_ and *β*_*xp*_ can be given as

(3) $$\begin{array}{c} \alpha_{xp}=-k_{p}d\sin\theta_{p}\cos\phi_{p}. \end{array}$$

In (3), *k*_*p*_ = 2*π*/*λ*_*p*_, where *λ*_*p*_ = *c*/*f*_*p*_. Similarly, *d* = *λ*_min⁡_/4, where *λ*_min⁡_ = *c*/*f*_max⁡_. So, (3) can be represented as

(4) $$\begin{array}{c} \alpha_{xp}=-\frac{\pi f_{p}}{2f_{\max}}\left(\sin\theta_{p}\cos\phi_{p}\right). \end{array}$$

In the same way,

(5) $$\begin{array}{c} \beta_{xp}=\frac{\pi d^{2}\left(1-\text{si}{\text{n}}^{2}\theta_{p}\cos^{2}\phi_{p}\right)}{\lambda_{p}r_{p}}, \end{array}$$

where (5) can be further rewritten as

(6) $$\begin{array}{c} \beta_{xp}=\frac{\pi f_{p}^{2}}{16f_{\max}r_{p}}\left(1-\text{si}{\text{n}}^{2}\theta_{p}\cos^{2}\phi_{p}\right). \end{array}$$

Similarly, in (2), *α*_*yp*_ and *β*_*yp*_ can be given as

(7) $$\begin{array}{c} \alpha_{yp}=-\frac{\pi f_{p}}{2f_{\max}}\left(\sin\theta_{p}\sin\phi_{p}\right),\\ \beta_{yp}=\frac{\pi f_{p}^{2}}{16f_{\max}r_{p}}\left(1-\text{si}{\text{n}}^{2}\theta_{p}\text{si}{\text{n}}^{2}\phi_{p}\right). \end{array}$$

By using (4) and ((6)-(7)) in (1) and (2), we get:

(8) wm,0=∑p=1Papej((π/fmax⁡)(−(mfp/2)(sinθpcos⁡ϕp)+(m2fp2/16rp)(1−sin2θpcos⁡2ϕp)))+ηm,0,w0,n=∑p=1Papej((π/fmax⁡)(−(nfp/2)(sinθpsinϕp)+(n2fp2/16rp)(1−sin2θpsin2ϕp)))+η0,n,

where *f*_*p*_ represents the frequency of *p*th source, while *f*_max⁡_ is the maximum frequency to be used. In vector form (8) can be collectively represented as

(9) $$\begin{array}{c} w=Ba+\eta , \end{array}$$

where **w**,  **η**,  **a**, and  **B** can be defined as

(10) w=[w−Q,0w−Q+1,0⋯w−1,0  w1,0w2,0⋯wQ−1,0wQ,0w0,0w0,−Qw0,−Q+1⋯w0,−1w0,1w0,2⋯w0,Q−1w0,Q]T,η=[η−Q,0η−Q+1,0⋯η−1,0η1,0η2,0⋯ηQ−1,0ηQ,0η0,0η0,−Qη0,−Q+1⋯η0,−1η0,1η0,2⋯η0,Q−1η0,Q]T,a=[a1a2⋯aP]T,B=[b(αx1,βx1,αy1,βy1),…,b(αxp,βxp,αyp,βyp),…,b(αxP,βxP,αyP,βyP)],

where

(11) b(αxp,βxp,αyp,βyp) =[ej[(−Q)αxp+(−Q)2βxp]  ,ej[(−Q+1)αxp+(−Q+1)2βxp],…,ej[−αxp+βxp],ej[αxp+βxp],…  ,ej[(Q−1)αxp+(Q−1)2βxp],ej[(Q)αxp+(Q)2βxp],1,ej[(−Q)αyp+(−Q)2βyp],ej[(−Q+1)αyp+(−Q+1)2βyp],…,ej[−αyp+βyp],ej[αyp+βyp],…,  ej[(Q−1)αyp+(Q−1)2βyp],ej[(Q)αyp+(Q)2βyp]]T.

From (8), one can see that the unknown parameters are *a*_*p*_,  *f*_*p*_,  *r*_*p*_,  *θ*_*p*_, and *ϕ*_*p*_ where *p* = 1,2,…, *P*. So, the problem in hand is to estimate these 5D parameters jointly and efficiently; before starting the problem, it is important to find out the dimension of the signal subspace from the received snapshots.

## 3. Signal Subspace Dimension

For this purpose, we used nonparametric technique:

(12) $$\begin{array}{c} w=Ba+\eta , \end{array}$$

where **a** is a *P* × 1 source vector, **B** is our (4*Q* + 1) × *P* array manifold matrix, and **η** is an AWGN vector with spectral matrix *σ*_*η*_^2^ *I*. The spectral matrix of **w** is given as

(13) $$\begin{array}{c} S_{w}\left(\omega \right)=BA\left(\omega \right)B^{H}+\sigma^{2}I=S_{s}\left(\omega \right)+S_{\eta }\left(\omega \right), \end{array}$$

where

(14) $$\begin{array}{c} A\left(\omega \right)=E\left[a\left(\omega \right)a^{H}\left(\omega \right)\right]. \end{array}$$

We expect that the signals are incoherent, so that the rank of **S**_*s*_(*ω*) is equal to the number of signals. Let the rank of **S**_*s*_(*ω*) be *P*; then eigendecomposition of **S**_**w**_(*ω*) is given as

(15) $$\begin{array}{c} S_{w}\left(\omega \right)=Q_{s}\Lambda_{s}Q_{s}^{H}+Q_{\eta }\Lambda_{\eta }Q_{\eta }^{H}, \end{array}$$

where

(16) Λs=diag⁡[σs12σs22⋯σsP2],Λη=diag⁡[ση2ση2⋯ση2] with  (4Q+1)−P elements,

**Q**_*s*_ has column vectors which are eigenvectors of **S**_*s*_(*ω*), and **Q**_*η*_ has column vectors which are eigenvectors of **S**_*n*_(*ω*). We expect the last (4*Q* + 1) − *P* eigenvalues representing noise to be the smallest and also equal. For finding the dimensions of two subspaces, we can use the following hypothesis [25]:

(17) LP=((4Q+1)−P)ln⁡×[{(1/((4Q+1)−P))∑l=P+14Q+1λl}(∏l=P+14Q+1λl)1/((4Q+1)−P)].

This numerator is the arithmetic mean of (4*Q* + 1) − *P* being the smallest eigenvalues, while denominator is their geometric mean. We start with *P* = 1, then *P* is chosen correctly, and then the last (4*Q* + 1) − *P* eigen values are the smallest and equal, making *L*_*P*_(*P*) = 0. After having found *P* by this test, we know exactly the number of signals; whether any of these signals is friend, foe, or indifferent is not the topic of concern for this paper.

## 4. Proposed Schemes

In this section, brief introduction and flow diagram are provided for IPA, PS, and GA.

### 4.1. Interior Point Algorithm (IPA)

Interior point methods (barrier methods) can be used for linear and nonlinear convex optimization problems. It uses either conjugate gradient step through a trust region or Newton step by using linear programming in order to get an optimum solution during each iteration [26]. The IPA has extensive applications and performs very well particularly in the presence of less local minima. However, its performance is superb even in the presence of more local minima when it is used as a local search optimizer with PSO or GA. For detailed applications and derivation of the algorithm, it is recommended that reader should see [27]. By observing such applications, in this work IPA is mainly used as a local search optimizer with GA.

### 4.2. Pattern Search (PS)

Pattern search was introduced by Hookes and Jeeves in 1961 which is gradient or derivative free technique and can be used for both local and global optimization problems. Basically, PS works on mesh which is defined according to some specific rules. If no improvement is achieved in cost function at the mesh points of current iteration, then the mesh is polished and the process is repeated. It has applications in many fields, such as signal processing and soft computing [28]. In this work, PS is also mainly used as a local search optimizer with GA in which the best chromosome achieved through GA is given as the starting point to PS.

### 4.3. Genetic Algorithm (GA)

GA is basically different from previously discussed algorithm (IPA and PS) and is applicable to a wide range of optimization problems. GA is more prominent and proficient algorithm than any other evolutionary computing technique due to its ease in conception and ease in implementation and more importantly less probable to get stuck in the presence of local optima. GA is being successfully applied to a wide range of applications from commerce to scientific research [29].

**Figure 2:**
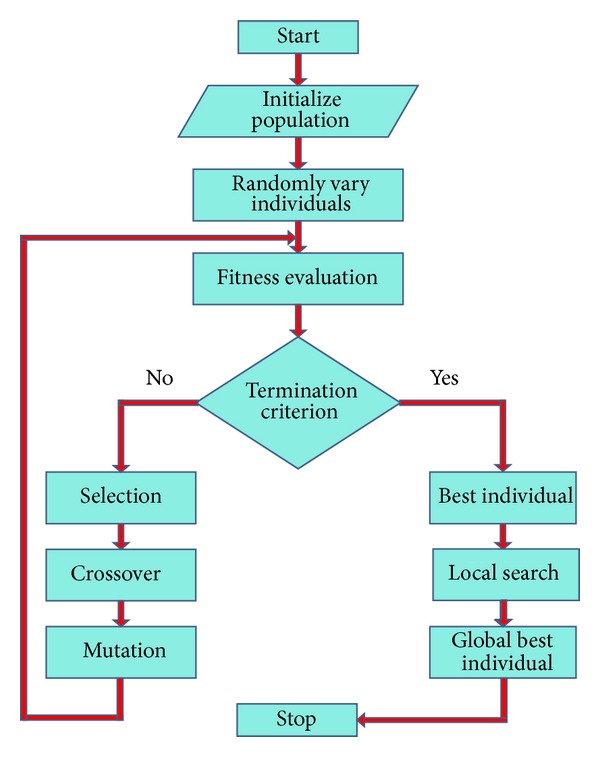
Flow diagram for hybrid GA-PS and GA-IPA.

The steps for GA and GA-PS in the form of pseudocode are given below, while their flow diagram is shown in Figure 2.

**Step 1 (initialization)**

In this step, we randomly generate *I* number of chromosomes, where the length of each chromosome is 5∗*P*. In each chromosome the first *P* genes represent amplitudes, the second *P* genes contain the frequencies, and the next *P* genes represent the ranges, while the fourth and fifth *P* genes represent elevation and azimuth angles, respectively, of the sources as follows:

(18) C=(a1,1a1,2⋯a1,P                f1,P+1f1,P+2⋯f1,2P  r1,2P+1r1,2P+2⋯  r1,3Pθ1,3P+1θ1,3P+2⋯θ1,4Pϕ1,4P+1ϕ1,4P+2⋯ϕ1,5Pa2,1a2,2⋯  a2,P      f1,P+1f1,P+2⋯f1,2Pr2,2P+1r2,2P+2⋯  r2,3P  θ2,3P+1θ2,3P+2⋯θ2,4Pϕ2,4P+1ϕ2,4P+2⋯ϕ2,5P⋮⋮⋮⋮aM,1aM,2⋯aM,P    f1,P+1f1,P+2⋯f1,2PrM,2P+1rM,2P+2⋯rM,3PθM,3P+1θM,3P+2⋯θM,4PϕM,4P+1ϕM,4P+2⋯ϕM,5P),

where

(19) $$\begin{array}{c} a_{in}\in R:L_{l}\leq a_{ip}\leq H_{l},\\ f_{i,P+p}\in R:f_{\min}\leq f_{i,P+p}\leq f_{\max},\\ r_{i,2P+p}\in R:L_{r}\leq r_{i,2P+p}\;\leq H_{r},\\ \theta_{i,3P+p}\in R:0\leq \theta_{i,3P+p}\leq \frac{\pi }{2},\\ \phi_{i,4P+p}\in R:0\leq \phi_{i,4P+p}\leq 2\pi ;\\ \text{for}\;\;i=1,2,\ldots M,\;p=1,2,\ldots P, \end{array}$$

where *L*_*l*_ and *l*_*r*_ are the lowest, while *H*_*l*_ and *H*_*r*_ are the highest limits of signals amplitude and range, respectively.

**Step 2 (fitness function)**

Our goal is to minimize the errors received for both subarrays. For *i*th chromosome, it can be given as

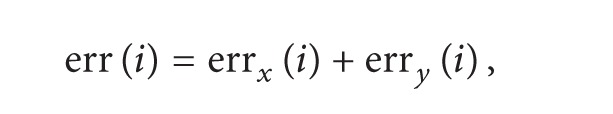
(20)

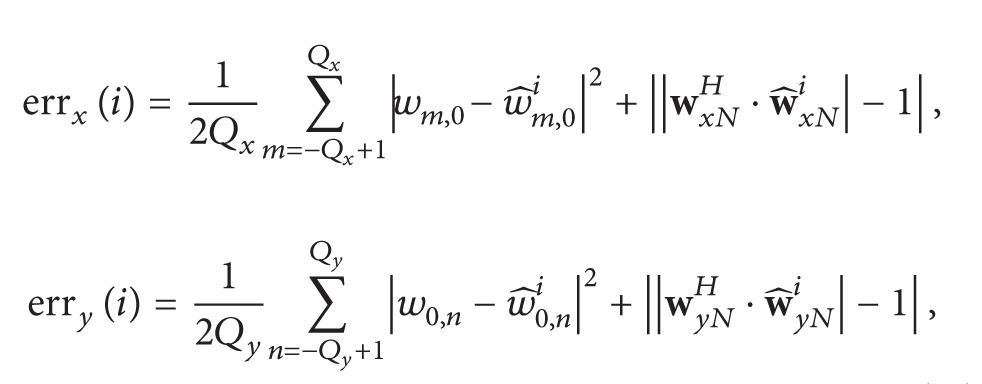
(21)
where in (21), *w*_*m*,0_ and *w*_0,*n*_ are defined in (8), respectively, while ${\hat{w}}_{m,0}^{i}$ and ${\hat{w}}_{0,n}^{i}$ are given as

(22) $$\begin{array}{c} {\hat{w}}_{m,0}^{i}=\overset{P}{\underset{p=1}{\sum }}{\hat{c}}_{p}^{i}e^{j(\left(\pi /f_{\max})(\left(-m({\hat{c}}_{P+p}^{i})/2)\sin({\hat{c}}_{3P+p}^{i})\cos({\hat{c}}_{4P+p}^{i})+(m^{2}{\left({\hat{c}}_{P+p}^{i}\right)}^{2}/16({\hat{c}}_{2P+p}^{i}))(1-\text{si}{\text{n}}^{2}({\hat{c}}_{3P+p}^{i})\cos^{2}({\hat{c}}_{4P+p}^{i})\right)\right))},\\ {{\hat{w}}^{i}}_{0,n}=\overset{P}{\underset{p=1}{\sum }}{{\hat{c}}^{i}}_{p}e^{j(\left(\pi /f_{\max})(\left(-n({\hat{c}}_{P+p}^{i})/2)\sin({\hat{c}}_{3P+p}^{i})\sin({\hat{c}}_{4P+p}^{i})+(n^{2}{\left({\hat{c}}_{P+p}^{i}\right)}^{2}/16({\hat{c}}_{2P+p}^{i}))(1-\text{si}{\text{n}}^{2}({\hat{c}}_{3P+p}^{i})\text{si}{\text{n}}^{2}({\hat{c}}_{4P+p}^{i})\right)\right))}. \end{array}$$

Similarly, in (21), $w_{xN},{{\hat{w}}^{i}}_{xN},w_{yN},\;\;\text{and}\;\;\;{{\hat{w}}^{i}}_{yN}$ can be defined as

(23) $$\begin{array}{c} w_{zN}=\frac{w_{z}}{||w_{z}||},\\ {{\hat{w}}^{i}}_{zN}=\frac{{{\hat{w}}^{i}}_{z}}{||{{\hat{w}}^{i}}_{z}||}, \end{array}$$

where *z* = *x*, *y*.

**Step 3 (termination criteria)**

The termination criteria depend on the following conditions if they are achieved:

- the objective function value is achieved which is predefined; that is, *ε* ≤ 10^−7^, or
- total number of iterations has been completed.

**Step 4 (reproduction)**

New population is reproduced by using the operators of crossover, elitism, and mutation selection as shown in Table 1.

**Step 5 (hybridization)**

In this important step, for further improvements, the best chromosome achieved through GA is given to PS and IPA as starting point. The parameter settings for IPA and PS are provided in Table 1.

**Step 6 (storage)**

For better statistical analysis, store the global best of the current run and repeat Steps 2–5 for sufficient numbers of independent runs.

## 5. Results and Discussion

In this section, several simulations are performed to validate the proposed schemes. Initially, the comparison of proposed hybrid schemes is carried out with the individual performance of GA, IPA, and PS in terms of estimation accuracy, convergence rate, and proximity effects. At the end of this section, the comparison of proposed schemes is made with the traditional existing technique [15] using error as a figure of merit. We have used a MATLAB built-in optimization tool box, for which the parameter settings are provided in Table 1. All the values of frequencies, ranges, and DOA are taken in terms of Mega-Hertz (MHz), wavelength (*λ*), and radians (rad), respectively. Every time, we have used same number of sensors in both subarrays, where the reference sensor is common for both. The interelement spacing between the two consecutive sensors in each subarray is taken as *λ*/4. Each result is averaged over 100 independent runs.

**Case 1**

In this case, the estimation accuracy of IPA, PS, GA, GA-IPA, and GA-PS is discussed for 2 sources. The CSCA consists of 9 sensors; that is, each subarray is composed of four sensors, while the reference sensor is common for them. In this case, no noise is added to the system. The desired values of amplitudes, frequencies, ranges, elevation, and azimuth angles are *a*_1_ = 6, *f*_1_ = 30 MHz, *r*_1_ = 2*λ*, *θ*_1_ = 0.2618 rad, and *ϕ*_1_ = 2.0071 rad; *a*_2_ = 4, *f*_2_ = 60 MHz, *r*_2_ = 0.6*λ*, *θ*_2_ = 1.1345 rad,  and *ϕ*_2_ = 2.9671 rad.

Although in this case, GA alone has produced fairly good estimation accuracy as provided in Table 2; however, it becomes even more accurate when hybridized with IPA and PS. Among all schemes, the GA-PS approach produced better results and maintained less error between desired values and estimated values. The second best scheme is GA-IPA, while GA alone provides the third best results.

**Case 2**

In this case, the estimation accuracy is discussed for 3 sources having values *a*_1_ = 3, *f*_1_ = 40 MHz, *r*_1_ = 2.5*λ*, *θ*_1_ = 0.4363  rad, and *ϕ*_1_ = 1.0472  rad; *a*_2_ = 1, *f*_2_ = 70 MHz, *r*_2_ = 5*λ*, *θ*_2_ = 0.7330  rad, and *ϕ*_2_ = 2.1817 rad; *a*_3_ = 7, *f*_3_ = 50 MHz, *r*_3_ = 0.2*λ*, *θ*_3_ = 1.3963  rad, and *ϕ*_3_ = 3.5779 rad. This time the array is composed of 13 sensors. Due to the increase of sources, the accuracy of IPA, PS, and GA has been significantly despoiled. However, as listed in Table 3, the accuracy of GA has improved when hybridized with IPA and PS.

The hybrid GA-PS technique proved to be the most accurate approach for three sources, while the second best approach is the other hybrid GA-IPA approach.

**Case 3**

In this case, the estimation accuracy of four near-field sources is discussed in the absence of noise where the CSCA is composed of 17 sensors. The desired values are *a*_1_ = 3.5, *f*_1_ = 65 MHz, *r*_1_ = 1*λ*, *θ*_1_ = 0.4712 rad,  and *ϕ*_1_ = 0.1745 rad; *a*_2_ = 5, *f*_2_ = 30 MHz, *r*_2_ = 6*λ*, *θ*_2_ = 0.8727 rad,  and *ϕ*_2_ = 2.0420  rad; *a*_3_ = 2, *f*_3_ = 85 MHz, *r*_3_ = 10*λ*,*θ*_3_ = 1.2741  rad,  and  *ϕ*_3_ = 2.7925 rad; *a*_4_ = 8, *f*_4_ = 25 MHz, *r*_4_ = 4*λ*, *θ*_4_ = 1.5184 rad, and *ϕ*_4_ = 4.4506 rad. One can see from Table 4 that the estimation accuracy of all schemes degraded as we have faced more local minima in this case. However, even in this case, the hybrid approaches especially the GA-PS performed well and made a close estimate of desired response. The second best scheme is again the other hybrid GA-IPA approach.

**Case 4**

**Figure 3:**
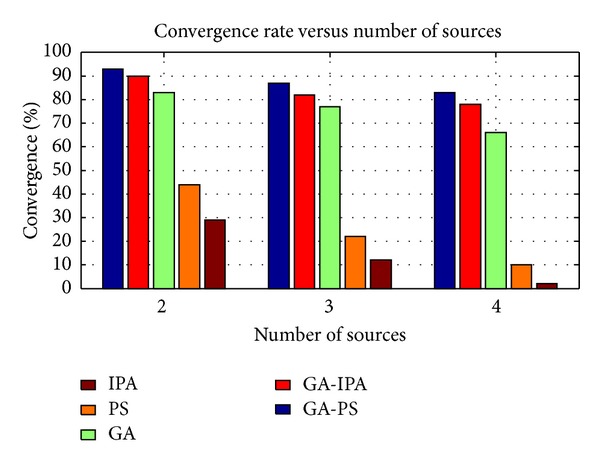
Convergence rate versus number of sources.

In Figure 3, convergence rate is shown for each scheme against different number of sources. From convergence, we mean, the total number of times a particular technique achieved its goal. In this case, we have taken the same two sources as given in Case 1, but this time the CSCA consists of 17 sensors for each number of sources. The bar graph shows that the hybrid GA-PS technique has converged many number of times as compared to the remaining approaches for all sources. The second best convergence rate is maintained by GA-IPA, while the third best scheme is GA alone.

**Case 5**

In this case, the estimation accuracy is checked in the presence of low signal to noise ratio (SNR). The value of SNR is 5 dB, while the array has 13 sensors. The desired values of amplitude, frequency, ranges, elevation and azimuth angles of 3 sources are *a*_1_ = 3, *f*_1_ = 70 MHz, *r*_1_ = 6*λ*, *θ*_1_ = 0.2618 rad,  and *ϕ*_1_ = 0.6109  rad; *a*_2_ = 1,*f*_2_ = 45 MHz, *r*_2_ = 2.4*λ*, *θ*_2_ = 0.7854 rad,  and *ϕ*_2_ = 2.4435 rad; *a*_3_ = 7, *f*_3_ = 30 MHz, *r*_3_ = 4.3*λ*, *θ*_3_ = 1.4835 rad,  and *ϕ*_3_ = 3.7525  rad. As provided in Table 5, due to low SNR' the accuracy of all schemes is despoiled. However, the hybrid GA-PS scheme is robust enough to produce better results even in the presence of low SNR. The second best result is produced by the other hybrid GA-IPA scheme.

**Case 6**

**Figure 4:**
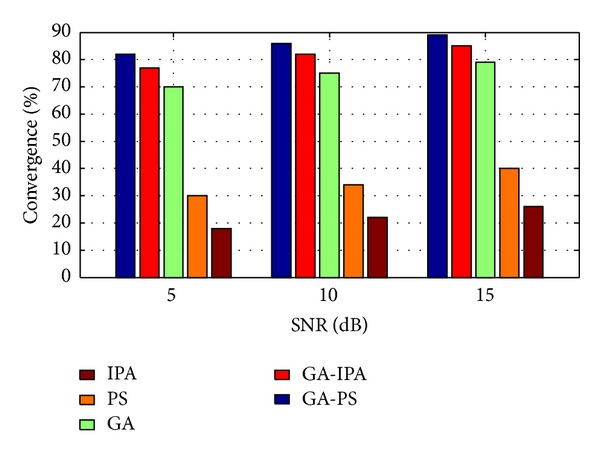
Convergence rate versus SNR.

In Figure 4, the convergence rate of each scheme is evaluated against noise and it has been shown that the convergence rate of all schemes degraded at low values of SNR. However, with the increase of SNR, the convergence rate of each scheme has improved. Again, the hybrid GA-PS has shown fairly good robustness against all the values of SNR.

**Case 7**

In this case, the proximity effect of DOA of three sources is evaluated in terms of estimation accuracy and convergence rate in the presence of 10 dB noise. As given in Table 6, due to proximity and low SNR, we have faced more local minima. However, once again one can see that the hybrid GA-PS produced fairly good results in terms of accuracy and convergence rate even in this case, while the second best result is given by GA-IPA.

**Case 8**

**Figure 5:**
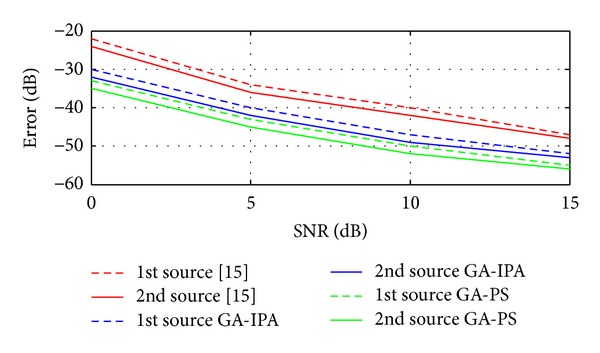
Error estimation of the frequencies versus SNR.

**Figure 6:**
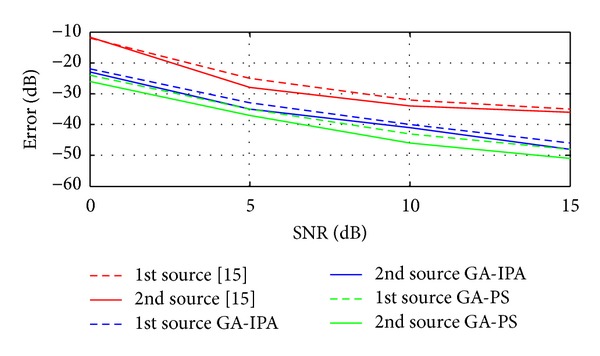
Error estimation of the azimuth angles versus SNR.

**Figure 7:**
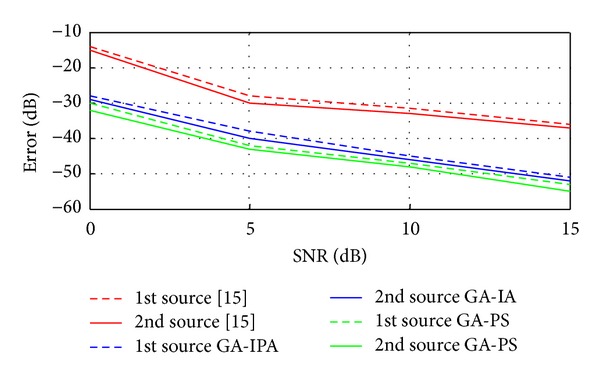
Error estimation of the elevation angles versus SNR.

**Figure 8:**
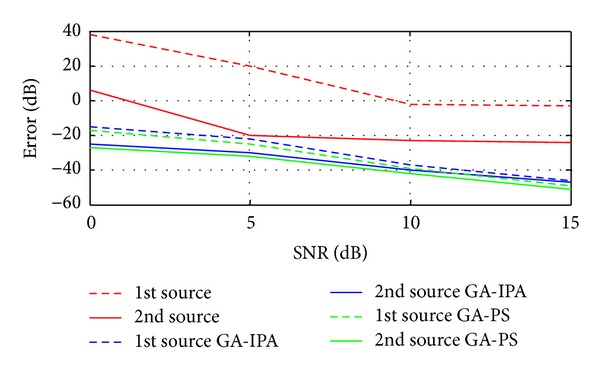
Error estimation of the ranges versus SNR.

**Figure 9:**
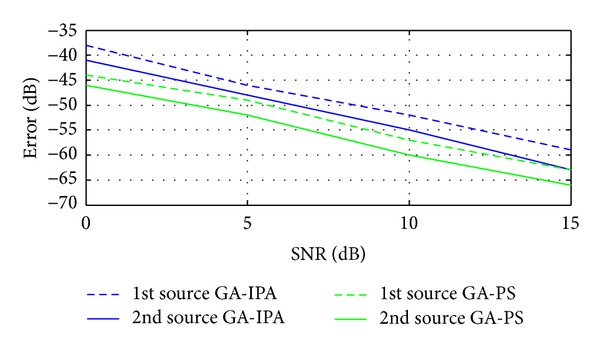
Error estimation of the amplitudes versus SNR.

In this case, we have compared the proposed two hybrid schemes with traditional technique given in [15]. Basically, in [15], Liang et al. have proposed a cumulants based technique to estimate the 4D parameters (Frequency, range, elevation angle, and azimuth angle) of near-field sources. In [15], mean square error (MSE) is used, while in the current work, the error is the combination of MSE and correlation between the desired and estimated vectors as discussed in Section 3. For these simulations, two sources are considered in the presence of noise. The values of the two sources are exactly the same as given above in Case 1. Figures 5, 6, 7, and 8 have shown the error for frequency, azimuth angle, elevation angle, and range of two near-field sources by using [15] and the two proposed hybrid schemes. One can clearly observe that, in each case (especially for range estimation), the proposed schemes have maintained fairly minimum error as compared to [15]. Besides, [15] is unable to estimate the amplitude, while our proposed schemes have shown satisfactory error for amplitude estimation as shown in Figure 9.

## 6. Conclusion and Future Work

In this work, we have mainly developed two hybrid schemes (GA-IPA and GA-PS) to estimate the 5D parameters (amplitude, frequency, range, elevation angle, and azimuth angle) of sources located in the near field of the sensors array. A new multiobjective fitness function was developed, which is the combination of MSE and correlation between normalized desired and normalized estimated vectors. It requires only single snapshot. The two hybrid schemes have shown good performance as compared to their individual responses in terms of estimation accuracy, convergence rate, and so forth. The proposed schemes have also shown good results as compared to traditional technique by using an error as a figure of merit. However, the hybrid GA-PS proved to be the best approach among them for the joint estimation of amplitude, frequency, range, elevation angle, and azimuth angle of near-field sources.

In future, one can check the same approach for null steering and beam steering in the field of adaptive beamforming.

## Figures and Tables

**Table 1:** Parameter setting for GA, PS, and IPA.

GA		PS		IPA	
Parameters	Settings	Parameters	Setting	Parameters	Setting
Population size	240	Starting point	Best chromosome achieved by GA	Starting point	Best chromosome achieved by GA

Number of generations	1000	Polling order	Consecutive	Subproblem algorithm	Idl factorization

Migration direction	Both ways	Maximum iteration	1000	Maximum perturbation	0.1

Crossover fraction	0.2	Function evaluation	17000	Minimum perturbation	1*e* ^−8^

Crossover	Heuristic	Mesh size	01	Scaling	Objective and constraint

Function tolerance	10–12	Expansion factor	2.0	Hessian	BFGS

Initial range	(0-1)	Contraction factor	0.5	Derivative type	Central difference

Scaling function	Rank	Penalty factor	100	Penalty factor	100

Selection	Stochastic uniform	Bind tolerance	10-04	Maximum function evaluation	50000

Elite count	2	Mesh tolerance	10-07	Maximum iteration	1000

Mutation function	Adaptive feasible	X tolerance	10-06	X tolerance	10–12

**Table 2:** Estimation accuracy of 2 sources using 9 sensors.

Scheme	*a*_1_	*f*_1_ (MHz)	*r*_1_ (*λ*)	*θ*_1_ (rad)	*ϕ*_1_ (rad)	*a*_2_	*f*_2_ (MHz)	*r*_2_ (*λ*)	*θ*_2_ (rad)	*ϕ*_2_ (rad)
Desired values	6.0000	30.0000	2.0000	0.2618	2.0071	4.0000	60.0000	0.6000	1.1345	2.9671
IPA	6.0096	33.5342	2.0095	0.2714	2.0168	4.0095	63.5376	0.6095	1.1442	2.9767
PS	6.0050	32.7908	2.0051	0.2668	2.0123	4.0049	62.7546.	0.6050	1.1396	2.9722
GA	6.0031	30.9765	2.0032	0.2649	2.0103	4.0032	60.9782	0.6030	1.1376	2.9703
GA-IPA	6.0020	30.2289	2.0019	0.2638	2.0091	4.0020	60.2415	0.6018	1.1367	2.9692
GA-PS	6.0007	30.1089	2.0008	0.2625	2.0078	4.0008	60.1063	0.6007	1.1352	2.9678

**Table 3:** Estimation accuracy of 3 sources using 13 sensors.

Scheme	*a*_1_	*f*_1_ (MHz)	*r*_1_ (*λ*)	*θ*_1_ (rad)	*ϕ*_1_ (rad)	*a*_2_	*f*_2_ (MHz)	*r*_2_ (*λ*)	*θ*_2_ (rad)	*ϕ*_2_ (rad)	*a*_3_	*f*_3_ (MHz)	*r*_3_ (*λ*)	*θ*_3_ (rad)	*ϕ*_3_ (rad)
Desired values	3.0000	40.0000	2.5000	0.4363	1.0472	1.0000	70.0000	5.0000	0.7330	2.1817	7.0000	50.0000	0.2000	1.3963	3.5779
IPA	3.0557	46.9871	2.5558	0.4920	1.1030	1.0557	64.3425	5.0558	0.7888	2.2376	7.0558	57.0123	0.2557	1.4522	3.6337
PS	3.0338	45.1204	2.5338	0.4702	1.0810	1.0338	75.8734	5.0339	0.7668	2.2154	7.0339	55.8693	0.2339	1.4301	3.6117
GA	3.0092	43.7894	2.5093	0.4456	1.0565	1.0092	66.5682	5.0093	0.7423	2.1908	7.0092	54.1298	0.2093	1.4057	3.5871
GA-IPA	3.0065	41.2187	2.5066	0.4428	1.0538	1.0065	71.2654	5.0067	0.7396	2.1883	7.0066	51.2879	0.2067	1.4029	3.5847
GA-PS	3.0024	40.8903	2.5022	0.4388	1.0497	1.0024	70.8931	5.0025	0.7356	2.1841	7.0021	50.7969	0.2022	1.3986	3.5802

**Table 4:** Estimation accuracy of 4 sources using 17 sensors.

Scheme	*a*_1_	*f*_1_ (MHz)	*r*_1_(*λ*)	*θ*_1_ (rad)	*ϕ*_1_ (rad)	*a*_2_	*f*_2_ (MHz)	*r*_2_(*λ*)	*θ*_2_ (rad)	*ϕ*_2_ (rad)	*a*_3_	*f*_3_ (MHz)	*r*_3_(*λ*)	*θ*_3_ (rad)	*ϕ*_3_ (rad)	*a*_4_	*f*_4_ (MHz)	*r*_4_(*λ*)	*θ*_4_ (rad)	*ϕ*_4_ (rad)
Desired	3.5000	65.0000	1.0000	0.4712	0.1745	5.0000	30.0000	6.0000	0.8727	2.0420	2.0000	85.0000	10.0000	1.2741	2.7925	8.0000	25.0000	4.0000	1.5184	4.4506
IPA	3.5989	75.0947	1.0989	0.5702	0.2735	5.0989	19.8975	6.0990	0.9718	2.1409	2.0990	95.8734	10.0990	1.3731	2.8916	8.0989	15.0081	4.0990	1.6176	4.5497
PS	3.5687	73.8971	1.5688	0.5400	0.2432	5.0688	22.8912	6.0689	0.9414	2.1108	2.0686	92.8714	10.0688	1.3427	2.8613	8.0687	32.8145	4.0685	1.5873	4.5194
GA	3.5197	70.1879	1.0198	0.4908	0.1941	5.0195	24.9765	6.0196	0.8923	2.0616	2.0194	90.0012	10.0195	1.2936	2.8121	8.0194	30.8156	4.0195	1.5379	4.4702
GA-IPA	3.5166	67.3215	1.0164	0.4879	0.1913	5.0166	32.7957	6.0167	0.8894	2.0586	2.0166	87.0123	10.0167	1.2908	2.8091	8.0166	27.9099	4.0167	1.5350	4.4674
GA-PS	3.5105	66.0469	1.0103	0.4818	0.1850	5.0105	31.1201	6.0106	0.8834	2.0526	2.0107	86.3459	10.0108	1.2847	2.8030	8.0105	26.5562	4.0104	1.5289	4.4620

**Table 5:** Estimation accuracy for 3 sources at SNR = 5 dB.

Scheme	*a*_1_	*f*_1_ (MHz)	*r*_1_ (*λ*)	*θ*_1_ (rad)	*ϕ*_1_ (rad)	*a*_2_	*f*_2_ (MHz)	*r*_2_ (*λ*)	*θ*_2_ (rad)	*ϕ*_2_ (rad)	*a*_3_	*f*_3_ (MHz)	*r*_3_ (*λ*)	*θ*_3_ (rad)	*ϕ*_3_ (rad)
Desired	3.0000	70.0000	6.0000	0.2618	0.6109	1.0000	45.0000	2.4000	0.7854	2.4435	7.0000	30.0000	4.3000	1.4835	3.7525
IPA	3.3711	78.8790	6.1712	0.4329	0.7820	1.4711	54.9876	2.5712	0.9567	2.6145	7.1710	39.8791	4.4711	1.6547	3.9235
PS	3.2047	77.2137	6.1047	0.3665	0.7156	1.3047	53.1124	2.1047	0.8901	2.5483	7.1045	38.1236	4.4048	1.5884	3.8572
GA	3.1824	75.8711	6.0423	0.3142	0.6534	1.2422	49.8879	2.4425	0.8279	2.4860	7.0426	35.4398	4.3424	1.5259	3.7950
GA-IPA	3.0584	72.3298	6.0385	0.3002	0.6494	1.1385	47.6675	2.4384	0.8239	2.4820	7.0382	32.9983	4.3384	1.5220	3.7910
GA-PS	3.0357	71.1903	6.0156	0.2775	0.6266	1.0958	46.1290	2.4158	0.8013	2.4592	7.0158	31.6722	4.3158	1.4993	3.7684

**Table 6:** Proximity effect of DOA of three sources and 17 sensors at SNR = 10 dB.

Scheme	*θ*_1_ (rad)	*ϕ*_1_ (rad)	*θ*_2_ (rad)	*ϕ*_2_ (rad)	*θ*_3_ (rad)	*ϕ*_3_ (rad)	% convergence
Desired values	0.6981	1.9199	0.7679	1.9897	0.8378	2.0595	—
IPA	0.8203	2.0402	0.8901	2.1118	0.9599	2.1817	1
PS	0.7941	2.0351	0.8849	2.1049	0.9512	2.1712	4
GA	0.7400	1.9600	0.8116	2.0298	0.8796	2.1031	64
GA-IPA	0.7208	1.9408	0.7906	2.0141	0.8587	2.0857	70
GA-PS	0.7103	1.9303	0.7821	2.0019	0.8482	2.0717	80
